# The Importance of Bone Mineral Density in Hip Arthroplasty: Results of a Survey Asking Orthopaedic Surgeons about Their Opinions and Attitudes Concerning Osteoporosis and Hip Arthroplasty

**DOI:** 10.1155/2016/8079354

**Published:** 2016-11-23

**Authors:** Gerrit Steffen Maier, Kristina Kolbow, Djordje Lazovic, Uwe Maus

**Affiliations:** University Hospital for Orthopaedic Surgery, Pius Hospital, Carl-von-Ossietzky University Oldenburg, Oldenburg, Germany

## Abstract

*Objective.* In patients scheduled to undergo total joint arthroplasty of the hip, the bone quality around the joint affects the safety of prosthetic implantation. Bone strength is clinically assessed by measuring bone mineral density (BMD); therefore we asked if BMD is important to orthopaedic surgeons performing hip arthroplasty.* Methods.* In a 14-question survey, we asked about treatment patterns with respect to BMD, osteoporosis work-up, and treatment for patients with low BMD scheduled to undergo hip arthroplasty.* Results.* 72% of all asked orthopaedics reported to use cementless implants as a standard in hip arthroplasty. Over 60% reported that low BMD is a reason to reconsider operation strategies, but only 4% performed BMD measurement preoperatively. 26% would change their treatment strategy in case of a BMD (T-Score) between −1.5 and −2 and 40% in case of a T-score between −2 and −2.5, and 29% would change their intraoperative strategy if a T-score smaller than −2.5 was measured.* Conclusion.* The majority of orthopaedic surgeons who responded to the survey reported that they do not perform routine measurement of BMD before arthroplasty. However, most surgeons commented that low bone mineral density will influence their surgical plan and the implant design.

## 1. Introduction 

Due to demographic changes, the elderly population has increased and orthopaedic surgeons are confronted with more and more elderly patients undergoing hip arthroplasty [1]. Commonly, osteoporosis and osteoarthritis coexist in the elderly and can affect their quality of life [2]. Several studies suggest a high prevalence of unknown osteoporosis in patients scheduled to undergo hip arthroplasty [3, 4]. Furthermore, the decreased bone quality in the elderly may be a problem for arthroplastic surgeries, including problems like intraoperative fracture, periprosthetic osteolyses with implant migration, and postoperative periprosthetic fracture [5]. Historically, primary choice for implant fixation in total hip arthroplasty in osteoporotic bone has been a cemented implant design [6]. But cementless techniques and short-stem design are gaining more and more popularity in hip arthroplasty due to the ease of surgical procedure and increasing concerns about medical consequences during the cementing process and the consequences of cement removal in case of revision arthroplasty [6, 7]. Unfortunately these were originally designed for patients with normal bone structure and normal healing capacity [8]. Osseointegration of uncemented components needs high initial stability to enable new bone ingrowth and ongrowth [9]. Therefore, poor bone quality may jeopardize the initial stability of cementless implants. The bone-implant interface needs to withstand high shear stresses of physiological loading, and poor peri-implant bone quality may be a risk for the long-term success of osteointegration [10]. Aro et al. compared migration of hydroxyapatite-coated femoral stems in 39 women with or without low systemic bone mineral density. Patients with low bone mineral density showed higher subsidence of the femoral stem than did those with normal bone mineral density (*p* = 0.05). Low systemic bone mineral density and low local hip bone mineral density were risk factors for delayed translational stability [8].

Bone mineral density (BMD) is a clinical marker for bone strength, usually assessed by dual-energy X-ray absorptiometry (DXA). Bone mineral density varies among different body regions [2]. The bone mineral density of the hip is of particular interest to orthopaedic surgeons, due to its association with a higher risk of hip fracture [11]. Several studies showed that low bone mineral density of the hip affects the longevity of prosthetic implants following total hip arthroplasty [12, 13]. Low bone mineral density in combination with high body mass index increases the risk of early femoral component failure following hip resurfacing arthroplasty [14].

The purpose of this study was to assess the awareness and report practice patterns of orthopaedic specialists performing hip arthroplasty on a regular level regarding bone mineral density and osteoporosis with emphasis on hip arthroplasty. It is of importance to understand orthopaedic surgeons' opinions and practice patterns regarding screening and treatment of osteoporosis and osteomalacia with respect to hip arthroplasty.

## 2. Methods

A fourteen-question survey was administered to several national and international orthopaedic associations in order to forward this questionnaire to their members. The survey asked about treatment patterns with respect to bone mineral density and osteoporosis in patients scheduled to undergo hip arthroplasty. The full version of the questionnaire can be found at https://de.research.net/s/BoneMineralDensity.

Initially, orthopaedic surgeons were asked what kind of implant design they routinely use in hip arthroplasty. Furthermore, we asked if a known osteoporosis in the patients history influences the decision making process which implant to use. Then we asked study participants if they routinely perform dual-energy X-ray absorptiometry (DEXA) imaging and/or metabolic bone laboratory (bone turnover markers, vitamin D, and parathyroid hormone) before performing arthroplastic surgeries. The respondents were then asked if low bone mineral density is a reason to reassess the implant choice. Respondents were further queried what* t*-score would make them reassess their implant choice, how they would react to low bone mineral density, and if they routinely refer patients with low bone mineral density for additional osteoporosis workup.

The following orthopaedic associations distributed the survey to their members: Swiss Orthopaedics (Switzerland), Österreichische Gesellschaft für Orthopädie und Orthopädische Chirurgie (Austria), Dachverband Osteologie (Germany/Switzerland/Austria), New Zealand Orthopaedic Association (New Zealand), Macedonian Association of Orthopaedics and Traumatology (Macedonia), and Estonian Orthopaedic Society (Estonia).

The sample population was comprised of orthopaedic surgeons performing hip arthroplasty on a regular level.

Statistical tests were performed using Excel (Microsoft Inc., Redmont WA, USA) and SPSS software (SPSS, SPSS Inc., Chicago, IL, USA). Both descriptive and inferential statistical methods were included in our analysis. The study population was described by calculating the frequencies and percentages for categorical variables, which were subsequently compared using the Chi-square test. Statistical significance was established at a *p* value smaller than 0.05.

## 3. Results

465 questionnaires were returned; 32 were excluded because of incomplete information or because of responding orthopaedic surgeon not performing hip arthroplasty on a regular level, resulting in a total of 433 completed surveys that were available for analysis. According to the biographical information that was collected we received answers from orthopaedic surgeons from Germany (*n* = 123, rate of response 26%), Switzerland (*n* = 121, rate of response 18%), Austria (*n* = 118, rate of response 15%), New Zealand (*n* = 56, rate of response 22%), Estonia (*n* = 37, rate of response 27%), and Macedonia (*n* = 20, rate of response 22%).

**Figure 1:**
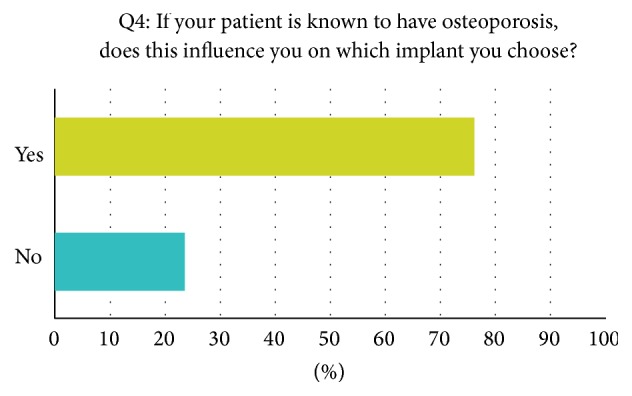
Example question taken from the survey, answers in percent.

**Figure 2:**
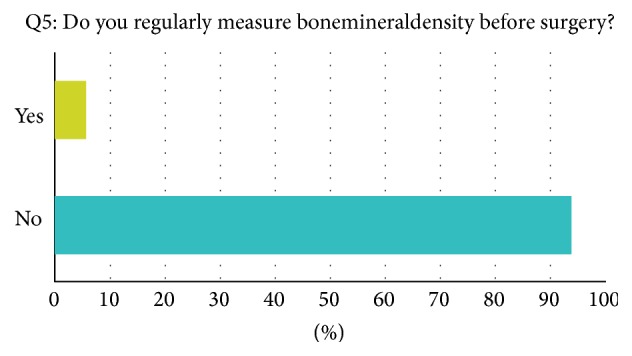
Example question taken from the survey, answers in percent.

**Figure 3:**
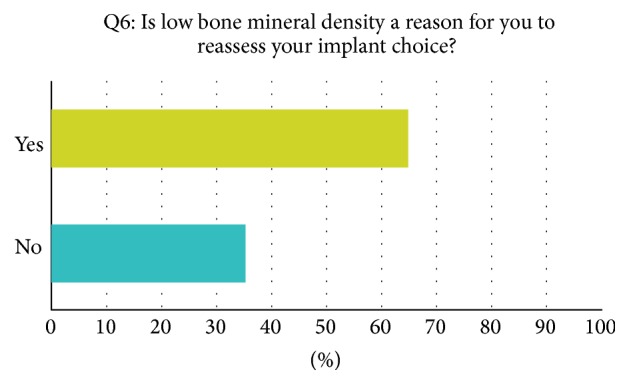
Example question taken from the survey, answers in percent.

A mean of 252 annually performed hip arthroplasties per institution was reported. Seventy-two percent of orthopaedic surgeons reported using cementless implants on a regular level. For 28 percent cemented implants are their first choice. Long-stem and short-stem implants are regularly used by 30% and 20% of all asked surgeons, respectively. When asked if a known osteoporosis in their patients' history would influence their implant choice, 77% of reporting orthopaedic surgeons answered yes and 23% would not react to a known osteoporosis (Figure 1). In case of a known osteoporosis, all reporting surgeons (100%) would use cemented prosthesis as their first choice implant. 23% would additionally use cemented long-stem implants. Four percent of surgeons reported obtaining a regular measurement of bone mineral density before surgery; 96% are not performing measurement of bone mineral density before arthroplastic surgeries (Figure 2). Interestingly, of the surgeons who reported not obtaining bone mineral measurement before surgery, 65 percent explained that low bone mineral density is a reason for reassessing the implant choice; 35% are not influenced by low bone mineral density (Figure 3). A* T*-score smaller than −2.5 is for 29 percent of reporting surgeons the personal cut-off value to reassess the operative strategy. 40% would start reassessment with* T*-scores between −2 and −2.5. A* T*-score between −1.5 and −2.5 is the personal cut-off value for 26% of all participating orthopaedic surgeons. In 99% of all answers, reassessment would result in the implantation of cemented prosthesis.

If intraoperatively the suspicion of osteoporosis arises, 76 percent of all asked surgeons would recommend diagnostic investigation in an outpatient clinic later on. 24% would start diagnostic investigation during the hospital stay. In 66% of all answers, diagnostic investigation is carried out by performing a dual-energy X-ray absorptiometry (DEXA) scan; 33 percent are performing quantitive computed tomography (Q-CT). Furthermore, 44% reported to additionally assess specific risk factors for osteoporosis and 17% of all asked orthopaedic surgeons are measuring bone turnover markers. If the diagnosis of osteoporosis is secured 42% of study participants would immediately start with antiosteoporotic therapy according to the national guidelines. Twenty-four percent reported to recommend starting with therapy according to the national guidelines. Thirty-five percent of asked surgeons reported just to inform the treating general practitioner about the diagnosis and to leave further treatment up to him.

## 4. Discussion

We demonstrated a high inconsistency in the treatment pattern of orthopaedic surgeons performing arthroplastic surgeries when they are confronted with the diagnosis or suspicion of osteoporosis or low bone mineral density. Despite upcoming developments in osteoporotic therapy and diagnosis, patients receiving total hip arthroplasty usually do so without a systemic evaluation of their bone health. In this recent study, ninety-six percent of all responding orthopaedic surgeons do not measure bone mineral density before performing total hip arthroplasty.

In the year 2000 Kanis et al. reported a prevalence of femoral neck osteoporosis in the Swedish population aged between 65 and 69 years to be 7.4% in males and 20.2% in females [15]. With advancing age, the percentage of patients with severe osteoporosis or osteopenia is believed to increase.

Historically, cemented stems have been preferred for femoral fixation in total hip replacement of the elderly. Cemented techniques provide strong and quick stability immediately after the implantation and do not require additional time for biologic fixation, enabling patients to be mobilised immediately after surgery [6]. Due to these results, cemented implant designs have been used with excellent results and with no particular long-term problems [16]. Due to the demographic change and the increasing age of patients undergoing total hip replacement, more cement-related problems have been reported. Issack et al. demonstrated that the reduced cardiovascular reserve in the advanced age inhibits a possible adaption to problems related to the cementation of implants, possibly leading to serious complications like fat embolism [7]. In an in-hospital mortality study of patients undergoing total hip replacement, mortality primarily occurred during the application of cement in patients with femoral neck fractures, highlighting the risks related to use of cement in these procedures [17]. These emerging concerns have led to cementless designs gaining more and more popularity in the orthopaedic community. But cementless implants have been designed for good bone health; low bone mineral density may have some major potential complications in cementless total hip arthroplasty. Increased migration and subsequent loss of the optimal stem position, delayed osseointegration of stem due to increased migration, late loosening of the implant due to mechanical failure of ingrown trabecular bone, and an increased risk of periprosthetic fracture have been described [8, 18]. Few studies have examined the concerns regarding the use of cementless designs in elderly patients. Rao et al. reported that although widely used because of their strong press-fit effect, wedge-shaped stems can cause initial subsidence with weight-bearing due to not obtaining biological fixation [19]. Bottner et al. demonstrated that young patients with good bone quality showed excellent results with weight-bearing immediately after surgery, in contrast to older or osteoporotic patients who needed to exercise in caution [20].

Seventy-two percent of all asked surgeons in this recent study are using cementless implants as their first choice implant in total hip replacement. In case of a known osteoporosis in the patients' history, 100% of the treating surgeons would not use cementless implants but cemented designs instead. Interestingly, for 65% of the surgeons who reported not to measure bone mineral density before arthroplasty, low bone mineral density would be a reason to reconsider their implant choice. 29% would reassess their op-strategy if their patient had a* t*-score smaller than −2.5; 40% would start to reassess with* T*-scores between −2 and −2.5. 26% of all responding orthopaedic surgeons would already start to reassess their choice of joint replacement design with* T*-scores between −1.5 and −2. 99% would use cemented implants after reassessment.

As described above, the decision between cemented or cementless implants for total hip replacement in the elderly is crucial. On the one hand there is a higher risk for cement-related complications, but on the other hand there is the risk of a delayed osseointegration and subsequent loss of the optimal stem position. Furthermore, the suspicion of low bone mineral density or osteoporosis often arises during the operation, confronting the operating surgeon with the question whether to reassess the operative strategy or not. Due to missing tools for an intraoperative diagnostic setup, orthopaedic surgeons often stay with their principal implant choice. When we asked orthopaedic surgeons what they would do if intraoperatively the suspicion of osteoporosis arises, 76% reported to recommend diagnostic investigations to be carried out in an outpatient clinic later on. Just twenty-four percent reported to start diagnostic investigation during the hospital stay. Furthermore, of these 24% of all asked surgeons, forty-two percent would immediately start antiosteoporotic therapy according to national guidelines. 35% of the corresponding surgeons would just inform the treating general practitioner about the diagnosis and leave further treatment up to him or her; 24% would at least recommend starting therapy according to the national guidelines. To determine why such a significant percentage of orthopaedic surgeons choose not to start with the medical therapy of osteoporosis remains a challenge. According to Skedros et al. this hesitancy could range from limited training in allowing other physicians the courtesy of dictating medication therapy. Further studies are needed to show the reason why many orthopaedic surgeons feel not responsible for medical therapy of osteoporosis [21, 22]. Simonelli et al. studied the treatment pattern of postmenopausal women admitted to hospital with a low-impact fracture. Although osteoporosis was diagnosed in these patients during their hospital stay, no antiosteoporotic medication was started. Even in the one-year follow-up examination no further antiosteoporotic treatment had been started. The message they received was that orthopaedic surgeons believed that the major responsibility of osteoporosis care lies with primary care physicians [23, 24]. Skedros et al. administered a 22-question survey to 171 orthopaedic surgeons in Utah, Idaho, and Wyoming with the purpose to determine the knowledge and opinions of orthopaedic surgeons with regard to their opportunities for initiating medical treatment of patients with an osteoporotic fracture. They concluded that although a majority of orthopaedists believe that they should expand their role in the medical osteoporotic therapy of patients with an osteoporotic fracture, many do not start medical treatment and believe that the patient's primary care provider has the responsibility to start with medical care [21].

There are several limitations to this study. Our survey required participants to assess their own practice patterns, resulting in a possible recall bias, which may affect the accuracy of the results. Furthermore, it is also conceivable that our study may be confounded by a selection bias because all surgeons were members of one of the abovementioned national or international orthopaedic associations, so it is unclear whether our results truly reflect the opinions and preferences of the orthopaedic community at large. Furthermore, response rate of the survey was quite low, which may lead to nonrepresentative results due to a selection bias. But we believe that the low response rate is a sign of the unpopularity of osteoporosis treatment in the orthopaedic community.

Not all orthopaedic surgeons may want to become knowledgeable in the treatment of osteoporosis. Hence, the field of bone health with regard to total joint replacement is still in its infancy. However, it is a field of growing importance to orthopaedic surgeons. An enhanced focus on bone health, for example, bone mineral density, holds several advantages, like reduced periprosthetic bone loss, prolongation of prosthetic lifetime, and reduced periprosthetic fractures. Due to the demographic change orthopaedic surgeons will be faced regularly with osteoporosis and osteomalacia while performing arthroplastic surgeries, resulting in an increasing need for awareness among orthopaedic surgeons regarding bone mineral density, osteoporosis screening, and treatment.
